# Interaction of Lamb Wave Modes with Weak Material Nonlinearity: Generation of Symmetric Zero-Frequency Mode

**DOI:** 10.3390/s18082451

**Published:** 2018-07-28

**Authors:** Xiaoqiang Sun, Xiangyan Ding, Feilong Li, Shijie Zhou, Yaolu Liu, Ning Hu, Zhongqing Su, Youxuan Zhao, Jun Zhang, Mingxi Deng

**Affiliations:** 1 College of Aerospace Engineering, Chongqing University, Chongqing 400044, China; Tiny_Strong@sina.com (X.S.); dingxiangyan@cqu.edu.cn (X.D.); feilong.li@cqu.edu.cn (F.L.); m18716480894@163.com (S.Z.); youxuan.zhao@cqu.edu.cn (Y.Z.); mejzhang@cqu.edu.cn (J.Z.); dengmx65@yahoo.com (M.D.); 2 Key Disciplines Lab of Novel Micro-nano Devices and System, International R & D Center of Micro-nano Systems and New Materials Technology, Chongqing University, Chongqing 400044, China; 3 Department of Mechanical Engineering, The Hong Kong Polytechnic University, Hong Kong SAR, China; zhongqing.su@polyu.edu.hk

**Keywords:** structural health monitoring, nonlinear Lamb waves, zero-frequency mode

## Abstract

The symmetric zero-frequency mode induced by weak material nonlinearity during Lamb wave propagation is explored for the first time. We theoretically confirm that, unlike the second harmonic, phase-velocity matching is not required to generate the zero-frequency mode and its signal is stronger than those of the nonlinear harmonics conventionally used, for example, the second harmonic. Experimental and numerical verifications of this theoretical analysis are conducted for the primary S_0_ mode wave propagating in an aluminum plate. The existence of a symmetric zero-frequency mode is of great significance, probably triggering a revolutionary progress in the field of non-destructive evaluation and structural health monitoring of the early-stage material nonlinearity based on the ultrasonic Lamb waves.

## 1. Introduction

Material non-destructive evaluation and structural health monitoring during the early stage of material degradation are crucial for structural integrity and safety [1]. The appearance of nonlinear effects in elastic wave propagation is one of the most notable and sensitive indicators for the onset of material damage [2,3,4,5,6]. Previous works have shown that the techniques based on nonlinear Lamb waves can be utilized for long-range and in-depth inspection [7,8,9] through characterizing acoustic nonlinearity, which in turn can be regarded as an indicator of the early-stage material nonlinearity caused by fatigue plasticity, precipitation hardening of thermal aging, corrosion, etc. [10,11,12,13,14]. In general, a nonlinear response is generated in the region where the early-stage material nonlinearity occurs and then several nonlinear wave components may appear including subharmonics or higher-order harmonics. All of these possible nonlinear components can be used to evaluate or monitor the material nonlinearity by exploiting their higher sensitivity to the changes of the material microstructure [15] compared with the conventional techniques based on linear waves [7,16]. In the research field of the nonlinear Lamb waves, the observation and utilization of the second harmonic is the focus [1,7,8,9,10,11,12,13,14,15,16,17,18,19,20,21,22,23,24,25,26]. However, an accurate experimental implementation of nonlinear Lamb waves is difficult due to the inherent multi-mode and dispersive nature. Also, the amplitude of the second harmonic of Lamb waves is cumulative only when certain conditions (phase-velocity matching, non-zero power flux or even group-velocity matching) are satisfied [7,25,27,28]. Furthermore, the intensity of these nonlinear wave components is generally low. Even when the signals are not submerged by noises, they are still difficult to be detected and estimated [2]. In fact, the propagation of an ultrasonic wave through a nonlinear material not only leads to the generation of the harmonics of the original wave but also the generation of a static displacement component (also called direct current component or zero-frequency mode) [29,30,31,32,33,34,35,36,37]. Narasimha et al. [34,35], Jacob et al. [36] and Nagy et al. [37] have shown that the zero-frequency mode varies linearly with the propagation distance. However, their studies are mainly confined to longitudinal acoustic waves.

To overcome these limitations, Sun et al. (theory) [38] and Wan et al. (simulation) [39] have shown that the signal of the zero-frequency mode is stronger than that of the traditional nonlinear harmonics for Lamb waves. Unlike the second harmonic, phase-velocity matching is not required for the zero-frequency mode accumulation. We theoretically, experimentally and numerically demonstrate the evidence, the efficiency and the feasibility of this concept in this paper.

**Figure 1:**
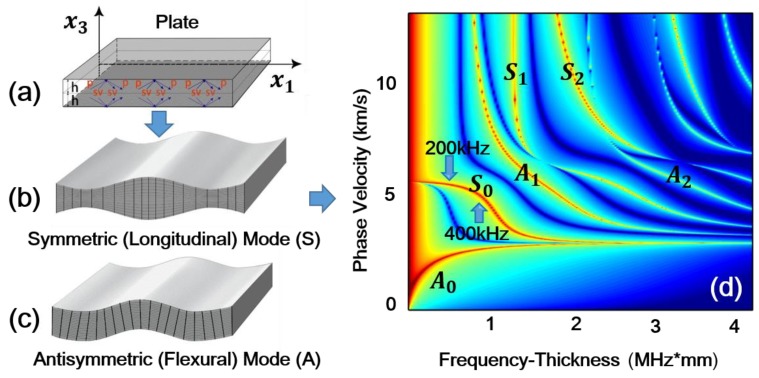
(Color online). Lamb waves in a plate problem. (**a**) Geometry of the plate which is infinite in the x_1_ direction. The reflections of the incident P- and SV-waves are illustrated. (**b**) Compressional waves in a plate (symmetric mode). (**c**) Flexural waves in a plate (antisymmetric mode). (**d**) Dispersion curves of Lamb waves in an aluminum plate: phase velocity.

The interaction of the elastic Lamb waves with a nonlinear plate medium is a fascinating and extremely complex process [5], during which a finite-amplitude wave propagates in an isotropic and homogeneous plate with stress-free surfaces. For simplicity, it is assumed that the wave propagates in the x_1_-direction and the particle motions are in the x_1_–x_3_ plane (Figure 1a). Geometrical and weak material nonlinearities are theoretically considered here and the total displacement field is assumed to be the sum of a primary wave $u_{\left(1\right)}$ (at frequency ω) and a second harmonic wave $u_{\left(2\right)}$ (at frequency 2 ω) based on the perturbation condition $\left\|u_{\left(2\right)}\right\|\ll \left\|u_{\left(1\right)}\right\|$ [15,21,22,40,41]. The perturbation method is used to solve the nonlinear motion equations for the harmonic generation in the waveguide. Solutions for the second harmonic, sum- and difference-frequency components are obtained via modal decomposition [28]. This procedure originally assumes that the nonlinearly generated secondary wave fields (perturbation solution) can be expressed as a superposition of the Lamb wave modes, since an orthogonality exists between different Lamb wave modes (completeness of Lamb wave modes is also assumed). It is obvious that the zero-frequency mode is orthogonal to other Lamb wave modes. Only when the zero-frequency mode is considered, the completeness of the mode expansion for the Lamb wave modes can be achieved. However, previous studies [15,21,25,28,40,41] ignored the solution for the zero-frequency mode theoretically. The present work firstly investigates the derivation of sum-frequency, difference-frequency, second harmonic and zero-frequency components of nonlinear Lamb waves in a plate with weak material nonlinearity. Then, the corresponding experimental and simulation results are demonstrated.

## 2. Formulations

### 2.1. Nonlinear Wave Equation

Following Lima et al. [21,28,40,41], the Green-Lagrange strain tensor, E, is related to the displacement tensor, u(X,t), by

(1) $$E=\frac{1}{2}(H+H^{T}+H^{T}H),\;H=\nabla u$$

where, X denotes the position of the material points in the reference configuration and H is the displacement gradient. Note that a black and bold letter denotes a tensor in this paper. The following strain energy, W(E), proposed by Landau and Lifshitz [42] for a nonlinear hyperelastic isotropic solid, is employed

(2) $$W(E)=\frac{1}{2}\lambda {\left[tr(E)\right]}^{2}+\mu tr(E^{2})+\frac{1}{3}C{\left[tr(E)\right]}^{3}+Btr(E)tr(E^{2})+\frac{1}{3}Atr(E^{3})$$

where, λ and μ are Lame’s constants, A, B and C denote the third-order elastic constants, which is related to material nonlinearity and tr[·] represents the trace of the bracketed tensor. The second Piola-Kirchhoff stress tensor, S, can be derived in terms of Lagrangian strain tensor, **E**, as

(3) $$S=\frac{\partial W(E)}{\partial E}=\lambda tr(E)I+2\mu E+C{\left[tr(E)\right]}^{2}I+Btr(E^{2})I+2Btr(E)E+AE^{2}$$

where, I is the identity tensor. Substitution of Equation (1) into (3) yields the second-order form of H,

(4) $$\begin{array}{c} S=\frac{\lambda }{2}tr(H+H^{T})I+\mu (H+H^{T})+\frac{\lambda }{2}tr(H^{T}H)I+\mu H^{T}H+C{\left[tr(H)\right]}^{2}I+\\ \frac{B}{2}tr(H^{2}+H^{T}H)I+Btr(H)(H+H^{T})+\frac{A}{4}(H^{2}+H^{T}H+HH^{T}+{\left(H^{T}\right)}^{2}) \end{array}$$

S can be decomposed into linear ($S^{L}$) and nonlinear ($S^{NL}$) components by

(5) $$\begin{array}{c} S=S^{L}+S^{NL},\;S^{L}=\lambda tr(H)I+\mu (H+H^{T}),\\ S^{NL}=\frac{\lambda }{2}tr(H^{T}H)I+\mu H^{T}H+C{\left[tr(H)\right]}^{2}I+\frac{B}{2}tr(H^{2}+H^{T}H)I\\ +Btr(H)(H+H^{T})+\frac{A}{4}(H^{2}+H^{T}H+HH^{T}+{\left(H^{T}\right)}^{2}). \end{array}$$

The equation of the wave motion is more conveniently written in terms of the first Piola-Kirchhoff stress tensor, T, which is related to the second Piola-Kirchhoff stress tensor, S, through the deformation gradient tensor: F=H+I, by

(6) $$T=F\cdot S=F\cdot (S^{L}+S^{NL})=(H+I)\cdot (S^{L}+S^{NL})$$

Then, T can also be decomposed into linear ($T^{L}(H)$) and nonlinear ($T^{NL}(H)$) components by

(7) $$\begin{array}{c} T(H)=T^{L}(H)+T^{NL}(H),\;T^{L}(H)=S^{L}=\lambda tr(H)I+\mu (H+H^{T}),\\ T^{NL}(H)=HS^{L}+S^{NL}=\frac{\lambda }{2}tr(H^{T}H)I+C{\left[tr(H)\right]}^{2}I+Btr(H)H^{T}+\frac{A}{4}{\left(H^{T}\right)}^{2}\\ +\frac{B}{2}tr(H^{2}+H^{T}H)I+(\lambda +B)tr(H)H+(\mu +\frac{A}{4})(H^{2}+H^{T}H+HH^{T}) \end{array}$$

where, only the terms up to the second-order are kept.

Considering the wave motion in an isotropic homogeneous plate (Figure 1a), the wave motion equation and stress-free boundary condition can be written as

(8) $$\nabla \cdot [T(H)]=\rho \ddot{u},\;{\left[T(H)\cdot n_{3}\right]|}_{x_{3}=\pm h}=0$$

where, $n_{3}$ is a unit vector parallel to the $x_{3}$ axis and 2h is the thickness of the plate as shown in Figure 1a.

Under the condition of weak nonlinearity, Equation (8) can be solved using a perturbation method through decomposing the wave field into the primary and secondary components

(9) $$u(X,t)=u_{\left(1\right)}(X,t)+u_{\left(2\right)}(X,t),\;\left\|u_{\left(2\right)}(X,t)\right\|\ll \left\|u_{\left(1\right)}(X,t)\right\|$$

where, $u_{\left(1\right)}(X,t)$ and $u_{\left(2\right)}(X,t)$ represent the primary and secondary wave field, respectively and ‖·‖ represents the amplitude of the corresponding wave field.

When substituting Equations (1), (7) and (9) into Equation (8), we obtain

(10) $$\begin{array}{c} \left(\lambda +2\mu )\nabla \nabla \cdot [u_{\left(1\right)}(X,t)+u_{\left(2\right)}(X,t)]-\mu \nabla \times \nabla \times [u_{\left(1\right)}(X,t\right)\\ +u_{\left(2\right)}(X,t)]+\nabla \cdot [T^{NL}(H)]=\rho [{\ddot{u}}_{\left(1\right)}(X,t)+{\ddot{u}}_{\left(2\right)}(X,t)],\\ {\left[T(H)\cdot n_{3}\right]|}_{x_{3}=\pm h}={\left[T^{L}(H)\cdot n_{3}+T^{NL}(H)\cdot n_{3}\right]|}_{x_{3}=\pm h}=0. \end{array}$$

Now, Equation (10) can be divided into two parts, that is, the first-order and second-order approximation parts. The first-order approximation is

(11) $$\begin{array}{c} (\lambda +2\mu )\nabla \nabla \cdot u_{\left(1\right)}(X,t)-\mu \nabla \times \nabla \times u_{\left(1\right)}(X,t)=\rho {\ddot{u}}_{\left(1\right)}(X,t),\\ {T^{L}[H(u_{\left(1\right)})]\cdot n_{3}|}_{x_{3}=\pm h}=0 \end{array}$$

which gives the primary wave field and the solution is

(12) $$\begin{array}{cc} \mathrm{SH}\;\mathrm{mode}: & q=\frac{n\pi }{2h},\left\{ \begin{array}{c} n\in \left\{0,2,4,\ldots \right\}\;\mathrm{symmteric},\\ n\in \left\{1,3,5,\ldots \right\}\;\mathrm{antisymmetric}, \end{array}\right.\\ \mathrm{RL}\;\mathrm{mode}: & \frac{\tan(qh)}{\tan(ph)}=-{\left[\frac{4k^{2}pq}{{\left(k^{2}-q^{2}\right)}^{2}}\right]}^{\pm 1},\left\{ \begin{array}{c} +1,\;\mathrm{symmteric},\\ -1,\;\mathrm{antisymmteric}, \end{array}\right.\\ & p^{2}=\frac{\omega^{2}}{c_{L}^{2}}-k^{2}\;\mathrm{and}\;q^{2}=\frac{\omega^{2}}{c_{T}^{2}}-k^{2} \end{array}$$

where, $c_{L}$ and $c_{T}$ are the velocities of the elastic longitudinal and transverse waves, respectively, SH and RL represent shear-horizontal and Rayleigh-Lamb, respectively. The second-order approximation is

(13) $$\begin{array}{c} (\lambda +2\mu )\nabla \nabla \cdot u_{\left(2\right)}(X,t)-\mu \nabla \times \nabla \times u_{\left(2\right)}(X,t)-\rho {\ddot{u}}_{\left(2\right)}(X,t)=-\nabla \cdot \left\{T^{NL}[H(u_{\left(1\right)},u_{\left(1\right)})]\right\},\\ {T^{L}[H(u_{\left(2\right)})]\cdot n_{3}=-T^{NL}[H(u_{\left(1\right)},u_{\left(1\right)})]\cdot n_{3}]|}_{x_{3}=\pm h} \end{array}$$

$T^{NL}[H(u_{\left(1\right)},u_{\left(1\right)})]$ and $\nabla \cdot \left\{T^{NL}[H(u_{\left(1\right)},u_{\left(1\right)})]\right\}$ which contain quadratic terms in $u_{\left(1\right)}$ can be taken as the force terms. Note that the higher-order force terms $T^{NL}[H(u_{\left(1\right)},u_{\left(2\right)})]$, $T^{NL}[H(u_{\left(2\right)},u_{\left(2\right)})]$, $\nabla \cdot \left\{T^{NL}[H(u_{\left(1\right)},u_{\left(2\right)})]\right\}$ and $\nabla \cdot \left\{T^{NL}[H(u_{\left(2\right)},u_{\left(2\right)})]\right\}$ are ignored. Then, Equation (13) can be regarded as a forced waveguide equation and be written as

(14) $$\begin{array}{c} (\lambda +2\mu )\nabla \nabla \cdot u_{\left(2\right)}(X,t)-\mu \nabla \times \nabla \times u_{\left(2\right)}(X,t)-\rho {\ddot{u}}_{\left(2\right)}(X,t)=-f^{\left(1,1\right)},\\ {{S^{\left(2\right)}\cdot n_{3}|}_{x_{3}=\pm h}=-S^{\left(1,1\right)}\cdot n_{3}|}_{x_{3}=\pm h}. \end{array}$$

where, $S^{\left(2\right)}=T^{L}[H(u_{\left(2\right)})]$, $S^{\left(1,1\right)}=T^{NL}[H(u_{\left(1\right)},u_{\left(1\right)})]$ and $f^{\left(1,1\right)}=\nabla \cdot \left\{T^{NL}[H(u_{\left(1\right)},u_{\left(1\right)})]\right\}$. The expressions for $S^{\left(1,1\right)}$ and $f^{\left(1,1\right)}$ can be derived by Equations (1) and (7) as

(15) $$\begin{array}{l} S_{ii}^{\left(1,1\right)}=\frac{\lambda }{2}\frac{\partial u_{(1)k}}{\partial x_{l}}\frac{\partial u_{(1)k}}{\partial x_{l}}+C\frac{\partial u_{(1)k}}{\partial x_{k}}\frac{\partial u_{(1)l}}{\partial x_{l}}+B\frac{\partial u_{(1)k}}{\partial x_{k}}\frac{\partial u_{(1)i}}{\partial x_{i}}+\\ \frac{B}{2}(\frac{\partial u_{(1)k}}{\partial x_{l}}\frac{\partial u_{(1)k}}{\partial x_{l}}+\frac{\partial u_{(1)k}}{\partial x_{l}}\frac{\partial u_{(1)l}}{\partial x_{k}})+\frac{A}{4}\frac{\partial u_{(1)i}}{\partial x_{k}}\frac{\partial u_{(1)k}}{\partial x_{i}}+(\lambda +B)\frac{\partial u_{(1)k}}{\partial x_{k}}\frac{\partial u_{(1)i}}{\partial x_{i}}+\\ (\mu +\frac{A}{4})(\frac{\partial u_{(1)i}}{\partial x_{k}}\frac{\partial u_{(1)i}}{\partial x_{k}}+\frac{\partial u_{(1)k}}{\partial x_{i}}\frac{\partial u_{(1)k}}{\partial x_{i}}+\frac{\partial u_{(1)i}}{\partial x_{k}}\frac{\partial u_{(1)k}}{\partial x_{i}})+o(u_{(1)i}{}^{3}),\\ S_{ij}^{\left(1,1\right)}=B\frac{\partial u_{(1)k}}{\partial x_{k}}\frac{\partial u_{(1)j}}{\partial x_{i}}+\frac{A}{4}\frac{\partial u_{(1)j}}{\partial x_{k}}\frac{\partial u_{(1)k}}{\partial x_{i}}+(\lambda +B)\frac{\partial u_{(1)k}}{\partial x_{k}}\frac{\partial u_{(1)i}}{\partial x_{j}}+\\ (\mu +\frac{A}{4})(\frac{\partial u_{(1)i}}{\partial x_{k}}\frac{\partial u_{(1)j}}{\partial x_{k}}+\frac{\partial u_{(1)k}}{\partial x_{i}}\frac{\partial u_{(1)k}}{\partial x_{j}}+\frac{\partial u_{(1)i}}{\partial x_{k}}\frac{\partial u_{(1)k}}{\partial x_{j}})+o(u_{(1)i}{}^{3}),\;\mathrm{for}\;i\neq j \end{array}$$

(16) $$\begin{array}{l} f_{i}^{\left(1,1\right)}=(\mu +\frac{A}{4})(\frac{\partial^{2}u_{(1)l}}{\partial x_{k}^{2}}\frac{\partial u_{(1)l}}{\partial x_{i}}+\frac{\partial^{2}u_{(1)l}}{\partial x_{k}^{2}}\frac{\partial u_{(1)i}}{\partial x_{l}}+2\frac{\partial^{2}u_{(1)i}}{\partial x_{l}\partial x_{k}}\frac{\partial u_{(1)k}}{\partial x_{l}})\\ +(\lambda +\mu +\frac{A}{4}+B)(\frac{\partial^{2}u_{(1)l}}{\partial x_{k}\partial x_{i}}\frac{\partial u_{(1)l}}{\partial x_{k}}+\frac{\partial^{2}u_{(1)l}}{\partial x_{l}\partial x_{k}}\frac{\partial u_{(1)i}}{\partial x_{k}})+(\lambda +B)\frac{\partial^{2}u_{(1)i}}{\partial x_{k}^{2}}\frac{\partial u_{(1)l}}{\partial x_{l}}\\ +(\frac{A}{4}+B)(\frac{\partial^{2}u_{(1)k}}{\partial x_{l}\partial x_{k}}\frac{\partial u_{(1)l}}{\partial x_{i}}+\frac{\partial^{2}u_{(1)l}}{\partial x_{i}\partial x_{k}}\frac{\partial u_{(1)k}}{\partial x_{l}})+(B+2C)\frac{\partial^{2}u_{(1)l}}{\partial x_{l}\partial x_{i}}\frac{\partial u_{(1)k}}{\partial x_{k}}+o(u_{(1)i}^{3}) \end{array}$$

### 2.2. Second-Order Solution

#### 2.2.1. Generation of Sum-Frequency, Difference-Frequency and Second Harmonic

Following de Lima et al. [21,28,40,41], consider two plane wave propagation modes with frequencies $\omega_{a}$ and $\omega_{b}$ and the corresponding wavenumbers $k_{a}$ and $k_{b}$, that are excited at $x_{1}=0$. The primary solution can be written as the form

(17) $$u_{\left(1\right)}(x_{1},x_{3},t)=\frac{1}{2}u_{\left(a\right)}(x_{3})e^{-i(k_{a}x_{1}-\omega_{a}t)}+\frac{1}{2}u_{\left(b\right)}(x_{3})e^{-i(k_{b}x_{1}-\omega_{b}t)}+c.c.$$

where, c.c. denotes the complex conjugate (Here, Equation (17) can also be written as $u_{\left(1\right)}=u_{\left(a\right)}(x_{3})\cos(k_{a}x_{1}-\omega_{a}t)+u_{\left(b\right)}(x_{3})\cos(k_{b}x_{1}-\omega_{b}t)$). Equation (17) can be introduced into Equations (15) and (16) to obtain

(18) $$\begin{array}{l} S^{\left(1,1\right)}=S_{a+a}^{\left(1,1\right)}(x_{3})e^{-i2(k_{a}x_{1}-\omega_{a}t)}+S_{b+b}^{\left(1,1\right)}(x_{3})e^{-i2(k_{b}x_{1}-\omega_{b}t)}+\\ S_{a+b}^{\left(1,1\right)}(x_{3})e^{-i[(k_{a}+k_{b})x_{1}-(\omega_{a}+\omega_{b})t]}+S_{a-b}^{\left(1,1\right)}(x_{3})e^{-i[(k_{a}-k_{b})x_{1}-(\omega_{a}-\omega_{b})t]}+c.c.\\ f=f_{a+a}^{\left(1,1\right)}(x_{3})e^{-i2(k_{a}x_{1}-\omega_{a}t)}+f_{b+b}^{\left(1,1\right)}(x_{3})e^{-i2(k_{b}x_{1}-\omega_{b}t)}+\\ f_{a+b}^{\left(1,1\right)}(x_{3})e^{-i[(k_{a}+k_{b})x_{1}-(\omega_{a}+\omega_{b})t]}+f_{a-b}^{\left(1,1\right)}(x_{3})e^{-i[(k_{a}-k_{b})x_{1}-(\omega_{a}-\omega_{b})t]}+c.c. \end{array}$$

where, $S_{a+a}^{\left(1,1\right)}(x_{3})$ and $f_{a+a}^{\left(1,1\right)}(x_{3})$ are determined by the self-interaction of the excited mode $u_{\left(a\right)}(x_{3})$, $S_{b+b}^{\left(1,1\right)}(x_{3})$ and $f_{b+b}^{\left(1,1\right)}(x_{3})$ are determined by the self-interaction of the excited mode $u_{\left(b\right)}(x_{3})$ and $S_{a+b}^{\left(1,1\right)}(x_{3})$, $S_{a-b}^{\left(1,1\right)}(x_{3})$, $f_{a+b}^{\left(1,1\right)}(x_{3})$ and $f_{a-b}^{\left(1,1\right)}(x_{3})$ are due to the mutual interaction of $u_{\left(a\right)}(x_{3})$ with $u_{\left(b\right)}(x_{3})$. The terms with respect to $e^{-i2(\omega_{a}+\omega_{b})t}$ and $e^{-i2(\omega_{a}-\omega_{b})t}$ are the sum- and difference-frequency components, respectively and the terms related to $e^{-i2\omega_{a}t}$ and $e^{-i2\omega_{b}t}$ are the second harmonic components.

According to [28], to simplify the notation, Equation (18) can be written as the following form

(19) $$\begin{array}{l} S^{\left(1,1\right)}=S_{\pm }^{\left(1,1\right)}(x_{3})e^{-i[(k_{a}\pm k_{b})x_{1}-(\omega_{a}\pm \omega_{b})t]}+c.c.,\\ f=f_{\pm }^{\left(1,1\right)}(x_{3})e^{-i[(k_{a}\pm k_{b})x_{1}-(\omega_{a}\pm \omega_{b})t]}+c.c. \end{array}$$

Following Auld [43] and de Lima and Hamilton [28], the secondary solution is derived as follows

(20) $$\begin{array}{cc} v_{\left(2\right)}(X,t) & =\frac{\partial u_{\left(2\right)}(X,t)}{\partial t}=\frac{\partial }{\partial t}[\frac{1}{2}\sum_{m=1}^{\infty }A_{m}(x_{1})u_{m}(x_{3})e^{-2i(\omega_{a}\pm \omega_{b})t}+c.c.],\\ & =\frac{1}{2}\sum_{m=1}^{\infty }a_{m}(x_{1})v_{m}(x_{3})e^{-2i(\omega_{a}\pm \omega_{b})t}+c.c.,\\ & \;4P_{mn}[\frac{d}{dx_{1}}+ik_{n}^{*}]a_{m}(x_{1})=f_{n}e^{-i(k_{a}\pm k_{b})x_{1}} \end{array}$$

where,

(21) $$\begin{array}{c} a_{m}(x_{1})=\frac{f_{n}}{4P_{mn}}\left\{ \begin{array}{cc} \frac{i}{\left[(k_{a}\pm k_{b})-k_{n}^{*}\right]}[e^{-i(k_{a}\pm k_{b})x_{1}}-e^{-ik_{n}^{*}x_{1}}], & \mathrm{for}\;k_{n}^{*}\neq k_{a}\pm k_{b}\\ x_{1}e^{-i(k_{a}\pm k_{b})x_{1}}, & \mathrm{for}\;k_{n}^{*}=k_{a}\pm k_{b} \end{array}\right.\\ P_{mn}\equiv -\frac{1}{4}\int_{-h}^{+h}(\frac{v_{n}^{*}}{2}\cdot \frac{T_{m}}{2}+\frac{v_{m}}{2}\cdot \frac{T_{n}^{*}}{2}\cdot )\cdot {\hat{x}}_{1}d{\hat{x}}_{3},\;P_{mn}=0\;\mathrm{if}\;k_{m}\neq k_{n}^{*},\\ f_{n}\equiv f_{n}^{surfce}+f_{n}^{volume},\;f_{n}^{surfce}\equiv \frac{1}{2}{[v_{n}^{*}(x_{3})\cdot S_{\pm }^{\left(1,1\right)}(x_{3})]\cdot {\hat{x}}_{3}|}_{x_{3}=-h}^{x_{3}=+h},\\ f_{n}^{volume}\equiv -\frac{1}{2}\int_{-h}^{+h}v_{n}^{*}(x_{3})\cdot f_{\pm }^{\left(1,1\right)}(x_{3})d{\hat{x}}_{3}\\ T_{m}\equiv \lambda \mathrm{tr}[\nabla u_{m}(x_{3})]I+\mu (\nabla u_{m}(x_{3})+u_{m}(x_{3})\nabla ) \end{array}$$

The subscript m and n represent the $m^{\mathrm{th}}$ and $n^{\mathrm{th}}$ modes at the frequency $\omega_{a}\pm \omega_{b}$, the superscript * denotes the complex conjugate of the corresponding physical variables, $v_{m}$ (short for $v_{m}(x_{3})$) is the particle velocity of the $m^{\mathrm{th}}$ mode and $T_{m}$, u, $S^{\left(1,1\right)}$ and $f^{1,1)}$ can also be expressed as a linear combination of the corresponding waveguide modes (not presented here) similar to that of v.

According to the above equation $P_{mn}=0\;\mathrm{if}\;k_{m}\neq k_{n}^{*},$ a propagating mode m is orthogonal to all modes except itself and $P_{mm}$ is the complex power flux of the $m^{\mathrm{th}}$ propagating mode in the direction ${\hat{x}}_{3}$. $f_{n}^{surfce}$ and $f_{n}^{volume}$ are interpreted as the power flux through the surface and the volume, respectively, due to the primary wave. When $k_{n}^{*}=k_{a}\pm k_{b}$ and $f_{n}\neq 0$, the amplitude of the secondary solution increases linearly in the direction of the propagation. Hence, two conditions must be satisfied for the internal resonance: (1) phase-velocity matching, $k_{n}^{*}=k_{a}\pm k_{b}$; (2) non-zero power flux, $f_{n}\neq 0$.

On the basis of Equation (20), the sum- and difference-frequency components can be generated through internal resonance and the second harmonic generation can be considered as a special case of the sum-frequency generation, in which only a single mode is excited. Then the solution for the second harmonic can be written in the normal mode expansion form [15,22,25].

(22) $$\begin{array}{c} v_{\left(2\right)}(x_{1},x_{3},t)=\sum_{n=1}^{\infty }a_{n}(x_{1})v_{n}(x_{3})e^{-i2\omega t}+c.c.\\ a_{n}(x_{1})=\frac{\left(f_{n}^{surface}+f_{n}^{volume}\right)}{\left(4P_{nn}\right)}\left\{ \begin{array}{ccc} i[e^{-i2kx_{1}}-e^{-ik_{n}^{*}x_{1}}]/(k_{n}^{*}-2k) & \mathrm{for} & k_{n}^{*}\neq 2k\\ x_{1}e^{-i2kx_{1}} & \mathrm{for} & k_{n}^{*}=2k \end{array}\right. \end{array}$$

where, $a_{n}(x_{1})$ is the amplitude of the n^th^ second harmonic mode $v_{n}(x_{3})$, k and $k_{n}^{*}$ are the wave numbers of the primary mode and the n^th^ second harmonic mode, respectively, $P_{nn}$ is the power carried by the nth second harmonic mode and $f_{n}^{volume}$ and $f_{n}^{surface}$ are power fluxes from the primary mode to the second harmonic mode through the volume and surface of the plate, respectively. The detailed expressions for $f_{n}^{volume}$, $f_{n}^{surface}$ and $P_{nn}$ are complex and extremely lengthy and will not be presented here.

#### 2.2.2. Symmetry Properties and Zero-Frequency Generation

According to [15], the following notations are introduced: S represents a unspecified, generic element of the set of symmetric functions in $x_{3}$, while A represents a generic, unspecified element of the set of antisymmetric functions in $x_{3}$. It is obvious that a derivation of a displacement component function with respect to $x_{3}$ changes the type of symmetry in $x_{3}$, while a derivation with respect to $x_{1}$ does not change the type of symmetry in $x_{3}$. Also, the following rules can be obtained easily:

(23) $$\begin{array}{c} S\cdot S=S,\;S\cdot A=A\cdot S=A,\;A\cdot A=S,\\ S+S=S,\;A+A=A \end{array}$$

Moreover, the parity of a symmetric mode can be represented as

(24) $${\left\{S\;0\;A\right\}}^{T}$$

Now, all possible secondary modes can be considered by evaluating $f_{n}^{surface}$ and $f_{n}^{surface}$ based on parity. The parity of H can be written as

(25) $$\begin{array}{c} \mathrm{SH}-S↔\mathrm{symmetric}\;\mathrm{shear}-\mathrm{horizontal}\;\mathrm{mode},\;\mathrm{SH}-A↔\mathrm{antisymmetric}\;\mathrm{shear}-\mathrm{horizontal}\;\mathrm{mode},\\ \mathrm{RL}-S↔\mathrm{symmetric}\;\mathrm{Rayleigh}-\mathrm{Lamb}\;\mathrm{mode},\;\mathrm{RL}-A↔\mathrm{antisymmetric}\;\mathrm{Rayleigh}-\mathrm{Lamb}\;\mathrm{mode},\\ H=H(\mathrm{SH}-S)+H(\mathrm{SH}-A)+H(\mathrm{RL}-S)+H(\mathrm{RL}-A),\\ H(\mathrm{SH}-S)=\left[\begin{array}{ccc} \begin{array}{c} 0\\ S_{a}+S_{b}+S_{a}^{*}+S_{b}^{*}\\ 0 \end{array} & \begin{array}{c} 0\\ 0\\ 0 \end{array} & \begin{array}{c} 0\\ A_{a}+A_{b}+A_{a}^{*}+A_{b}^{*}\\ 0 \end{array} \end{array}\right],H(\mathrm{SH}-A)=\left[\begin{array}{ccc} \begin{array}{c} 0\\ A_{a}+A_{b}+A_{a}^{*}+A_{b}^{*}\\ 0 \end{array} & \begin{array}{c} 0\\ 0\\ 0 \end{array} & \begin{array}{c} 0\\ S_{a}+S_{b}+S_{a}^{*}+S_{b}^{*}\\ 0 \end{array} \end{array}\right],\\ H(\mathrm{RL}-S)=\left[\begin{array}{ccc} \begin{array}{c} S_{a}+S_{b}+S_{a}^{*}+S_{b}^{*}\\ 0\\ A_{a}+A_{b}+A_{a}^{*}+A_{b}^{*} \end{array} & \begin{array}{c} 0\\ 0\\ 0 \end{array} & \begin{array}{c} A_{a}+A_{b}+A_{a}^{*}+A_{b}^{*}\\ 0\\ S_{a}+S_{b}+S_{a}^{*}+S_{b}^{*} \end{array} \end{array}\right],H(\mathrm{RL}-A)=\left[\begin{array}{ccc} \begin{array}{c} A_{a}+A_{b}+A_{a}^{*}+A_{b}^{*}\\ 0\\ S_{a}+S_{b}+S_{a}^{*}+S_{b}^{*} \end{array} & \begin{array}{c} 0\\ 0\\ 0 \end{array} & \begin{array}{c} S_{a}+S_{b}+S_{a}^{*}+S_{b}^{*}\\ 0\\ A_{a}+A_{b}+A_{a}^{*}+A_{b}^{*} \end{array} \end{array}\right]. \end{array}$$

Note that the complex conjugate components are considered here. Hence the parity of $S^{\left(1,1\right)}(x_{3})$ and $f^{\left(1,1\right)}(x_{3})$ is determined as follows

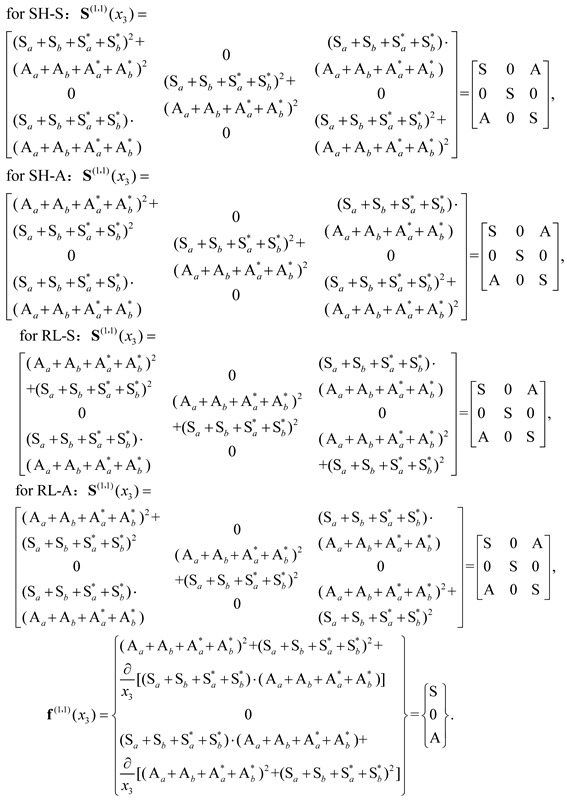
(26)

Then, $f_{n}^{surface}$ and $f_{n}^{surface}$ can be evaluated and only two terms below holding non-zero have been obtained

(27a) $$f_{n}^{surface}(\mathrm{RL}-S)={\frac{1}{2}\left\{\begin{array}{c} S(S_{a}+S_{b}+S_{a}^{*}+S_{b}^{*})\cdot (A_{a}+A_{b}+A_{a}^{*}+A_{b}^{*})+A\cdot \\ \left[{\left(A_{a}+A_{b}+A_{a}^{*}+A_{b}^{*}\right)}^{2}+(S_{a}+S_{b}+S_{a}^{*}+S_{b}^{*}{)}^{2}\right] \end{array}\right\}|}_{x_{3}=-h}^{x_{3}=+h}={\frac{1}{2}A|}_{x_{3}=-h}^{x_{3}=+h}\neq 0,$$

(27b) $$\begin{array}{c} f_{n}^{volume}(\mathrm{RL}-S)=-\frac{1}{2}\int_{-h}^{+h}\left\{\begin{array}{c} S\left\{\begin{array}{c} {\left(A_{a}+A_{b}+A_{a}^{*}+A_{b}^{*}\right)}^{2}+(S_{a}+S_{b}+S_{a}^{*}+S_{b}^{*}{)}^{2}+\\ \frac{\partial }{x_{3}}[(S_{a}+S_{b}+S_{a}^{*}+S_{b}^{*})\cdot (A_{a}+A_{b}+A_{a}^{*}+A_{b}^{*})] \end{array}\right\}+\\ A\left\{\begin{array}{c} (S_{a}+S_{b}+S_{a}^{*}+S_{b}^{*})\cdot (A_{a}+A_{b}+A_{a}^{*}+A_{b}^{*})+\frac{\partial }{\partial x_{3}}\\ \left[{\left(A_{a}+A_{b}+A_{a}^{*}+A_{b}^{*}\right)}^{2}+(S_{a}+S_{b}+S_{a}^{*}+S_{b}^{*}{)}^{2}\right] \end{array}\right\} \end{array}\right\}d{\hat{x}}_{3}\\ =-\frac{1}{2}\int_{-h}^{+h}Sdx_{3}\neq 0 \end{array}$$

Summarizing Equation (27), it is concluded that both a symmetric and an antisymmetric primary mode can excite a symmetric secondary mode and a symmetric zero-frequency mode. In contrast, neither a symmetric nor an antisymmetric primary mode can excite an antisymmetric secondary mode and an antisymmetric zero-frequency mode. This conclusion is similar to that of a previous study [15]. However, their conclusions are limited to the second harmonic generation only. Here, from Equation (27), we can see that ${\left(S_{a}\right)}^{2}$, ${\left(S_{b}\right)}^{2}$, ${\left(S_{a}^{*}\right)}^{2}$, ${\left(S_{b}^{*}\right)}^{2}$, ${\left(A_{a}\right)}^{2}$, ${\left(A_{b}\right)}^{2}$, ${\left(A_{a}^{*}\right)}^{2}$, ${\left(A_{b}^{*}\right)}^{2}$, $S_{a}A_{a}$, $S_{b}A_{b}$, $S_{a}^{*}A_{a}^{*}$ and $S_{b}^{*}A_{b}^{*}$ drive the second harmonic frequency; $S_{a}S_{b}$, $S_{a}A_{b}$, $S_{b}A_{a}$, $A_{a}A_{b}$, $S_{a}^{*}S_{b}^{*}$, $S_{a}^{*}A_{b}^{*}$, $A_{a}^{*}S_{b}^{*}$ and $A_{a}^{*}A_{b}^{*}$ drive the sum-frequency; $S_{a}S_{b}^{*}$, $S_{a}A_{b}^{*}$, $S_{b}A_{a}^{*}$, $S_{b}S_{a}^{*}$, $A_{a}A_{b}^{*}$, $A_{b}A_{a}^{*}$, $A_{b}A_{a}^{*}$ and $A_{b}S_{a}^{*}$ drive the difference-frequency; $S_{a}S_{a}^{*}$, $S_{a}A_{a}^{*}$, $S_{b}S_{b}^{*}$, $S_{b}A_{b}^{*}$, $A_{a}A_{a}^{*}$, $A_{a}S_{a}^{*}$, $A_{b}A_{b}^{*}$ and $S_{b}^{*}A_{b}$ drive the zero-frequency.

#### 2.2.3. Zero-Frequency Generation for a Primary Wave of a Single Mode

In order to obtain the solution of zero-frequency mode, one single mode is considered for simplicity. We write the primary displacement wave filed at frequency ω:

(28) $$u_{\left(1\right)}(x_{1},x_{3},t)=\frac{1}{2}u_{\left(1\right)}(x_{3})e^{-i(kx_{1}-\omega t)}+\frac{1}{2}u_{\left(1\right)}(x_{3})e^{i(kx_{1}-\omega t)}$$

Here, Equation (28) can also be written as $u_{\left(1\right)}(x_{1},x_{3},t)=u_{\left(1\right)}(x_{3})\cos(kx_{1}-\omega t)$.

Hence, H has the form

(29) $$H=\frac{1}{2}\left[\begin{array}{ccc} \begin{array}{c} -iku_{\left(1\right)}{}_{1}(x_{3})\\ -iku_{(1)2}(x_{3})\\ -iku_{(1)3}(x_{3}) \end{array} & \begin{array}{c} 0\\ 0\\ 0 \end{array} & \begin{array}{c} \frac{du_{(1)1}(x_{3})}{dx_{3}}\\ \frac{du_{(1)2}(x_{3})}{dx_{3}}\\ \frac{du_{(1)3}(x_{3})}{dx_{3}} \end{array} \end{array}\right]e^{-i(kx_{1}-\omega t)}+\frac{1}{2}\left[\begin{array}{ccc} \begin{array}{c} iku_{(1)1}(x_{3})\\ iku_{(1)2}(x_{3})\\ iku_{(1)3}(x_{3}) \end{array} & \begin{array}{c} 0\\ 0\\ 0 \end{array} & \begin{array}{c} \frac{du_{(1)1}(x_{3})}{dx_{3}}\\ \frac{du_{(1)2}(x_{3})}{dx_{3}}\\ \frac{du_{(1)3}(x_{3})}{dx_{3}} \end{array} \end{array}\right]e^{i(kx_{1}-\omega t)}$$

According to the procedure of Section 2.2.2, after ignoring the terms of the second harmonic components and setting $k_{a}=k_{b}=k,k_{n}^{*}=0,$ we obtain $P_{nn}^{0}$, $f_{n}^{0surface}$ and $f_{n}^{0volume}$ for Rayleigh-Lamb (RL) mode wave as

(30) $$P_{nn}^{0}\equiv -\frac{1}{4}\int_{-h}^{+h}(\frac{v_{n}^{*}}{2}\cdot \frac{T_{n}}{2}+\frac{v_{n}}{2}\cdot \frac{T_{n}^{*}}{2}\cdot )\cdot {\hat{x}}_{1}d{\hat{x}}_{3}=-\frac{1}{8}\int_{-h}^{+h}(v_{ni}T_{ni})d{\hat{x}}_{3},$$

(31) $$f_{n}^{0surface}={\left\{\begin{array}{l} \frac{1}{2}v_{n1}(x_{3})[(\frac{2B+A+2\mu }{4})k^{2}u_{(1)1}u_{(1)3}+(\frac{4\lambda +4B+8\mu +A}{8})u_{(1)1,3}u_{(1)3,3}]+\\ v_{n3}(x_{3})[(\frac{\lambda +2C+2B}{4}){\left[ku_{(1)1}\right]}^{2}+(\frac{2\lambda +2B+4\mu +A}{8})\left\{{\left[ku_{(1)3}\right]}^{2}+{\left[u_{(1)1,3}\right]}^{2}\right\}\\ +(\frac{3\lambda +2C+6\mu +6B+2A}{4}){\left[u_{(1)3,3}\right]}^{2} \end{array}\right\}|}_{x_{3}=-h}^{x_{3}=+h},$$

(32) $$f_{n}^{0volume}=-\frac{1}{2}\int_{-h}^{+h}\left\{\begin{array}{l} v_{n1}(x_{3})\left\{\begin{array}{l} \left(\frac{2B+2\mu +A}{4})k^{2}[u_{(1)1,3}u_{(1)3}+u_{(1)1}u_{(1)3,3}\right]\\ \left(\frac{4\lambda +4B+8\mu +A}{8})[u_{(1)1,33}u_{(1)3,3}+u_{(1)1,3}u_{(1)3,33}\right] \end{array}\right\}+\\ v_{n3}(x_{3})\left\{\begin{array}{l} \left(\frac{\lambda +2C+2B}{2})[ku_{(1)1}u_{(1)1,3}]+(\frac{2\lambda +2B+4\mu +A}{4}\right)\\ \left\{\begin{array}{c} k^{2}u_{(1)3}u_{(1)3,3}\\ +u_{(1)1,3}u_{(1)1,33} \end{array}\right\}+(\frac{3\lambda +2C+6\mu +6B+2A}{2})[u_{(1)3,33}] \end{array}\right\} \end{array}\right\}d{\hat{x}}_{3}$$

where, for RL-S mode,

(33) $$\begin{array}{l} v_{1}=-i\omega [ikA_{2}cos(px_{3})+B_{1}q\cos(qx_{3})]\\ v_{3}=-i\omega [-A_{2}p\sin(px_{3})-ikB_{1}sin(qx_{3})]\\ T_{1}=\mu [-2ikA_{2}p\sin(px_{3})+(k^{2}-q^{2})B_{1}sin(qx_{3})]\\ T_{3}=[-\lambda (k^{2}+p^{2})A_{2}cos(px_{3})-2\mu (p^{2}A_{2}cos(px_{3})+ikqB_{1}\cos(qx_{3}))] \end{array}\}$$

(34) $$\begin{array}{l} u_{(1)1}=\left\{\left[ikA_{2}cos(px_{3})+B_{1}q\cos(qx_{3})\right]\right\}\\ u_{(1)3}=\left\{\left[-A_{2}p\sin(px_{3})-ikB_{1}sin(qx_{3})\right]\right\} \end{array}\}$$

and for RL-A mode,

(35) $$\begin{array}{l} v_{1}=-i\omega [ikA_{1}sin(px_{3})-B_{2}q\sin(qx_{3})]\\ v_{3}=-i\omega [A_{1}p\cos(px_{3})-ikB_{2}cos(qx_{3})]\\ T_{1}=\mu [2ikA_{1}p\cos(px_{3})+(k^{2}-q^{2})B_{2}cos(qx_{3})]\\ T_{3}=[-\lambda (k^{2}+p^{2})A_{1}sin(px_{3})-2\mu (p^{2}A_{1}sin(px_{3})-ikqB_{2}\sin(qx_{3}))] \end{array}\}$$

(36) $$\begin{array}{l} u_{(1)1}=\left\{\left[ikA_{1}sin(px_{3})-B_{2}q\sin(qx_{3})\right]\right\}\\ u_{(1)3}=\left\{\left[A_{1}p\cos(px_{3})-ikB_{2}cos(qx_{3})\right]\right\} \end{array}\}$$

where, $A_{1}$, $A_{2}$, $B_{1}$ and $B_{2}$ are constants [44]. Hence, the amplitude of the zero-frequency mode is obtained as (by setting $k_{a}=k_{b}=k,\;k_{n}^{*}=0$ in Equation (20))

(37) $$a_{n}^{0}(x_{1})=(f_{n}^{0surface}+f_{n}^{0volume})x_{1}/4P_{nn}^{0}$$

where, $f_{n}^{0volume}$ and $f_{n}^{0surface}$ are power fluxes from the primary mode to the zero-frequency mode through the volume and surface of the plate, respectively and $P_{nn}^{0}$ is the power carried by the zero-frequency mode. However, the expressions for $P_{nn}^{0}$, $f_{n}^{0surface}$ and $f_{n}^{0volume}$ are complex and extremely lengthy and will not be presented here.

#### 2.2.4. Zero-Frequency Mode versus the Second Harmonic

According to Equations (22) and (37), the amplitude of the zero-frequency mode can grow linearly in the direction of the wave propagation and the condition of phase-velocity matching ($k_{n}^{*}=2k$) is no longer required (or the condition of phase-velocity matching is always satisfied). Only one condition for the accumulation of the symmetric zero-frequency mode is quantified, that is, the non-zero power flux.

The corresponding explicit solutions of Equations (22) and (37) are very complex. Therefore, the relationship between the amplitudes of the zero-frequency mode and the second harmonic is implicitly evaluated here. From Equations (22) and (37), we can write a sum amplitude of the zero-frequency mode and the second harmonic as $A_{n}(x_{1})=a_{n}^{0}(x_{1})+a_{n}(x_{1})$ = $g(H^{T}H,HH^{T})$. By considering a one-dimensional system in $x_{1}$ (simplified as x) and then, $H^{T}H={\left(\frac{du}{dx}\right)}^{2}$ and $HH^{T}={\left(\frac{du}{dx}\right)}^{2}$, so we have $A_{n}(x)=g({\left(\frac{du}{dx}\right)}^{2})\propto {\left(\frac{du}{dx}\right)}^{2}$. By only considering the second-order approximation, g is a linear function of ${\left(\frac{du}{dx}\right)}^{2}$ by neglecting the high-order terms. An approximate estimation can be simply made by setting u=sin(kx+ωt) and then ${\left(\frac{du}{dx}\right)}^{2}=k^{2}\cos^{2}(kx+\omega t)=\frac{k^{2}}{2}+\frac{k^{2}}{2}\cos2(kx+\omega t)$. The constant term, that is, $\frac{k^{2}}{2}$, is related to the zero-frequency mode and the other term, $\frac{k^{2}}{2}\cos2(kx+\omega t)$, denotes the second harmonic. Although this coefficient of the two wave modes is equal, the energy carried by the zero-frequency mode is two times larger than that of the second harmonic via integral operation. If the higher harmonics generated in this process are considered, more energy will flow into the zero-frequency mode while less energy will flow to the second harmonic. Based on the above analysis, we can conclude that the signal intensity of the zero-frequency mode should be much stronger than that of the second harmonic.

## 3. Experiment

**Figure 2:**
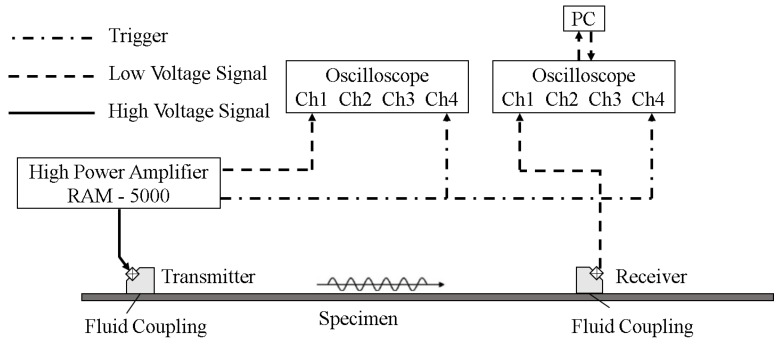
Schematic of experimental setup.

Figure 2 is a schematic of the experimental setup. The experimental study was carried out on a rectangular aluminum plate (2500 mm × 68 mm × 2 mm). Due to inevitable dislocation, micro-voids and multi-poles during the manufacture process of the aluminum plate, the specimen had weak material nonlinearity and no additional damage was introduced in the experiments. The Ritec SNAP system (RAM 5000) with a high power gated amplifier was used. A Hanning windowed tone burst signal of 200 kHz (the carrier frequency), 10 cycles and 100 Vp-p amplitude was input to the transmitting transducer with a center frequency of 250 kHz, which consisted of a Plexiglas wedge and a z-cut piezoelectric (LiNbO3) wafer. The carrier frequency 200 kHz is in the bandwidth of this transmitting transducer. The desired S0 Lamb wave can be induced by the refraction of the bulk longitudinal wave from the Plexiglas wedge into the aluminum plate. Note that, for simplicity, we focused on S0 mode working as the primary wave (Figure 1b). Guided waves travelling from the transmitter to the receiver (a Plexiglas wedge and a z-cut piezoelectric (LiNbO3) wafer) over a certain distance were measured. The center frequency of the receiver is 500 kHz. The received signal was amplified by both a preamplifier with −20 dB gain and a receiver amplifier with 32 dB gain.

**Figure 3:**
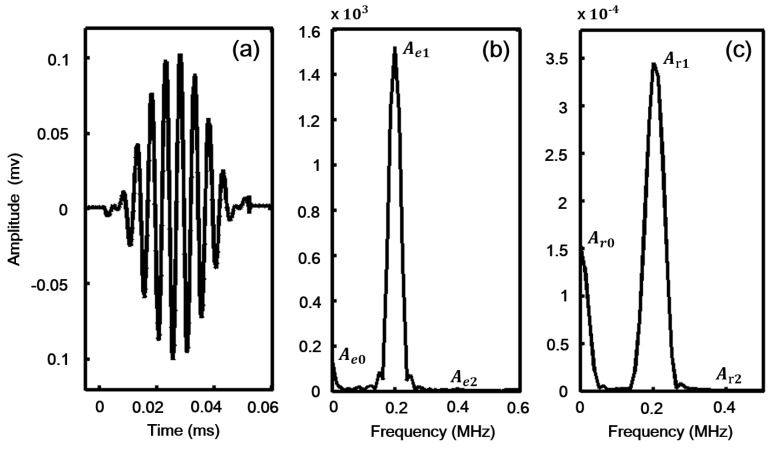
Some typical experimental signals: (**a**) Time domain (excitation signal). (**b**) Frequency domain (excitation signal). (**c**) The FFT result of the experiment signal received at 450 mm in the frequency-domain.

**Figure 4:**
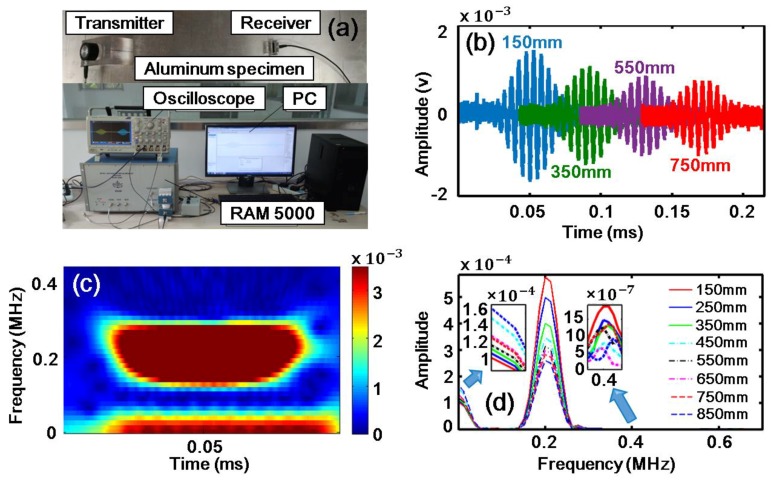
(Color online). Experiments. (**a**) Experiment setup and an aluminum plate specimen. (**b**) Experiment signals received at 150, 350, 550 and 750 mm in time-domain. (**c**) The STFT result of the wave packet at 150 mm within 0.1 ms. (**d**) The FFT results of the experiment signals received at 150, 250, 350, 450, 550, 650, 750 and 850 mm in the frequency-domain.

Typical experimental excitation signal in time and frequency domain are shown in Figure 3a,b. Noise analysis will be shown in Section 5. And typical experimental signals in the time-domain at the propagation distance of 150, 350, 550 and 750 mm are shown in Figure 4b, respectively. The result of the short-time Fourier transform (STFT) corresponding to the wave packet at 150 mm within 0.1 ms is demonstrated in Figure 4c, from which the components of both zero-frequency and the fundamental frequency can be identified clearly, whereas the second harmonic is too weak to be observed clearly. The results of the fast Fourier transform (FFT) are shown in Figure 4d, corresponding to eight propagation distances. The second harmonic and the zero-frequency mode were generated. The second harmonic is too weak to be observed at all signal extraction points. Again, the intensity of the zero-frequency mode is much stronger than that of the second harmonic. As the wave propagation distance increases, the amplitude of the zero-frequency mode increases while the amplitude of the fundamental wave decreases. The cumulative effect of the second harmonic is not obvious due to no strict matching of phase-velocity (see Figure 1d). Then, the energy of the primary wave is mainly transferred to the zero-frequency mode during the wave propagation.

## 4. Simulation

Numerical simulations on the propagation of Lamb waves in an aluminum plate with 2 mm thickness were also carried out using a commercial FEM software, that is, Abaqus, without considering the damping effect. Similar to [45], the Landau and Lifshitz model of hyper-elasticity was adopted by a user subroutine VUMAT, as generally used for material definition. The material properties are shown in Table 1. The simulation model of the plate is set to 2400 mm long, which is long enough to eliminate the reflected wave. The fixed boundary condition is applied to the right end to eliminate effect of rigid displacement during the simulation and the upper and lower surfaces of the plate are free. Prescribed uniform displacement is actuated on the left end of the plate to excite the desired primary S0 mode Lamb waves. The actuating function of the excitation signal is described as: $x(t)=\frac{A}{2}\sin(2\pi ft)(1-\cos(2\pi \frac{f}{N}t)$, where, *f* (=200 kHz) is the central frequency, *N* (=10) is the number of sinusoidal cycles in a pulse and *A* (=0.0001 mm) is the amplitude of tone-burst. Nineteen signal detection points are considered with a distance of 150, 250, 350, 450, 550, 650, 750, 850, 950, 1050, 1150, 1250, 1350, 1450, 1550, 1650, 1750, 1850 and 1950 mm from the left of the model, respectively.

**Figure 5:**
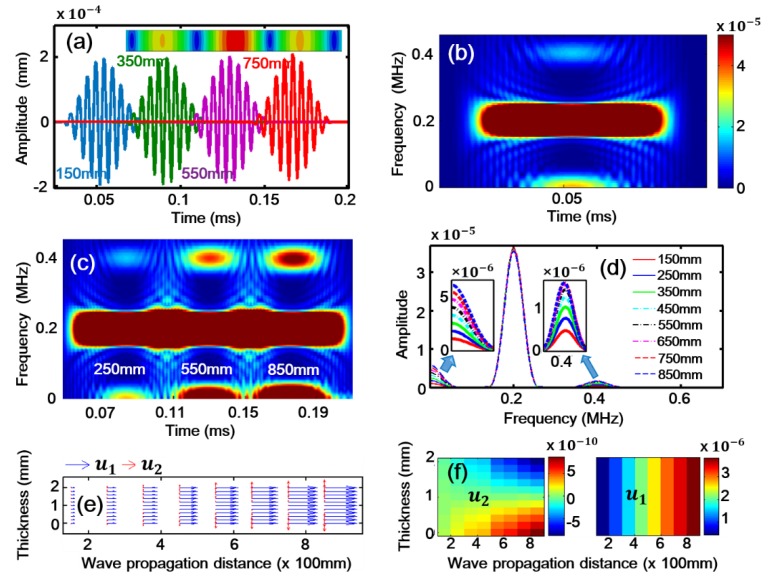
(Color online). Numerical simulations. (**a**) Simulation signals received at 150, 350, 550 and 750 mm in the time-domain. (**b**) The STFT results of the wave packet at 150 mm within 0.1 ms. (**c**) The STFT results of the wave packets at 250, 550 and 850 mm. (**d**) The FFT results of the simulation signals received at 150, 250, 350, 450, 550, 650, 750 and 850 mm in the frequency-domain. (**e**) The displacement fields vector graph of u_1_ and u_2_. (**f**) The displacement fields colored nephogram of u_1_ and u_2_.

Figure 5a shows the simulation results, that is, the wave packets at the propagation distances of 150, 350, 550 and 750 mm, respectively. The insert of Figure 5a illustrates the total displacement field including all frequency components, indicating a typical symmetrical deformation mode of the plate during wave propagation. The STFT result of the wave packet at 150 mm within 0.1 ms is demonstrated in Figure 5b, which is consistent with the experimental data shown in Figure 4c. In Figure 5b, the components of the zero-frequency, fundamental frequency and second harmonic can be observed, while the intensity of the second harmonic is much lower than those of the zero-frequency and fundamental frequency. Figure 5c shows the results of STFT at wave propagation distance 250, 550 and 850 mm, from which the intensity growth of the zero-frequency and second harmonic components with the distance can be identified, indicating their intrinsic cumulative characteristic. The FFT results of the wave packets at eight propagation distances are shown in Figure 5d. The intensity growth of the zero-frequency and second harmonic components can still be observed. Again, we can see that the intensity of the zero-frequency mode is much higher than that of the second harmonic. The above numerical results are basically consistent with the experimental data shown in Figure 4, though the material and damping properties of the experimental plate are different from those in the numerical model. In fact, the stronger zero-frequency mode compared with the second harmonic was also obtained in [45] through a COMSOL simulation; nevertheless, it was ignored. The results of our simulation are consistent with those of a previous study [39]. Physically, the zero-frequency displacement mode represents the irreversible shift of a particle from its original equilibrium position. To qualitatively pick up the displacement components of the zero-frequency mode, the following operation was performed. For all particles in a section along the through-thickness direction (nodes in FEM simulation), when a complete wave packet passing through this section, we discretized the wave packet into many discrete points in the time-domain and made the summation of u_1_ (along x_1_-axis in Figure 1a) and u_2_ (along x_3_-axis in Figure 1a). For all sections along the x_1_-axis, that is, the different wave propagation distances, we repeated the above operation and obtained the displacement fields for zero-frequency mode. The results of the displacement fields for u_1_ and u_2_ are shown in Figure 5e,f, indicating the symmetry of the zero-frequency mode. Moreover, u_1_ and u_2_ are accumulated as the propagation distance increases. This characteristic is schematically demonstrated in Figure 5e. We note in passing that, by varying the amplitude and the frequency of the excitation signal, the trends of the obtained results are basically the same, indicating the high reliability of the above observed phenomena.

## 5. Acoustic Nonlinearity Parameter

**Figure 6:**
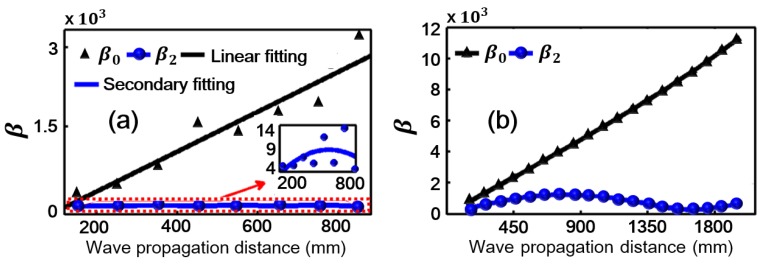
(Color online). Acoustic nonlinearity parameter. (**a**) β, a measure of material nonlinearity, plotted as a function of propagation distance for zero-frequency mode ($\beta_{0}$) and the second harmonic ($\beta_{2}$) (experiment). (**b**) β plotted as a function of propagation distance for zero-frequency mode ($\beta_{0}$) and the second harmonic ($\beta_{2}$) (simulation).

**Figure 7:**
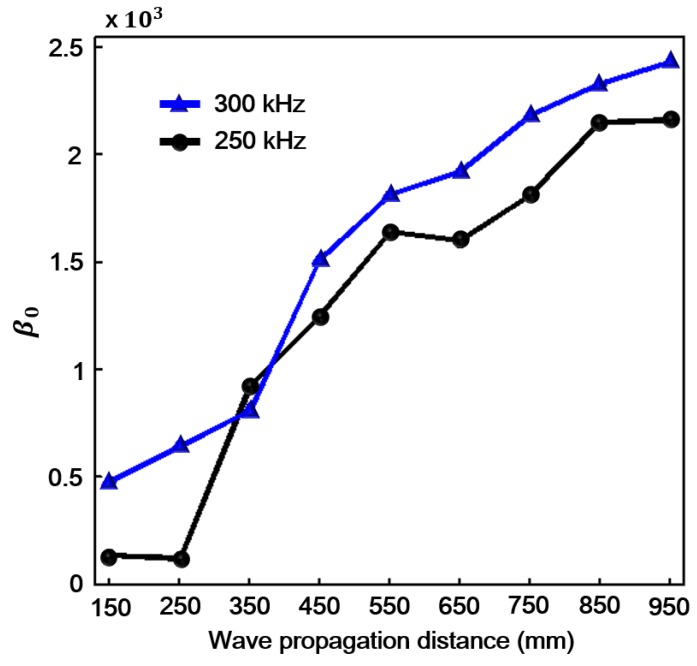
Acoustic nonlinearity parameter $\beta_{0}$ plotted as a function of propagation distance for 250 and 300 kHz (experiment).

According to previous studies [7,8,9,10,12,13,14,18,19,20,23,24], the measured acoustic nonlinearity parameter of Lamb waves can be expressed as $\beta \propto A_{2}/A_{1}^{2}$, where, $A_{1}$ and $A_{2}$ are the measured amplitudes of the fundamental and the second harmonic signals, respectively. Here, the expressions $\beta_{0}\propto A_{0}/A_{1}^{2}$ and $\beta_{2}\propto A_{2}/A_{1}^{2}$ are adopted to measure the acoustic nonlinearity, where, $A_{0}$ is the measured amplitude of the zero-frequency mode. It can be seen from Figure 3b that the experimental excitation signal contains an obvious zero-frequency component and a weak second harmonic component. This is caused by electro-instrument noises such as circuit quadratic nonlinearity, the contact nonlinearity between transducers (transmitter and piezoelectric wafer sensors) and specimen. In order to reduce the influences of these noises, the following data processing method was adopted. We set the amplitudes of zero-frequency, fundamental frequency and second harmonic of excitation signal as $A_{e0}$, $A_{e1}$ and $A_{e2}$ respectively, as shown in Figure 3b. For each received signal, the amplitudes of zero-frequency, fundamental frequency and second harmonic were represented as $A_{r0}$, $A_{r1}$ and $A_{r2}$ respectively, as shown in Figure 3c. Then the amplitudes of zero-frequency (second harmonic) of the received signal were rescaled using the equation $A_{r0}^{*}=A_{r0}-A_{e0}A_{r1}/A_{e1}$ ($A_{r2}^{*}=A_{r2}-A_{e2}A_{r1}/A_{e1}$). Finally, the acoustic nonlinearity parameter could be calculated by the equation $\beta_{0}=A_{r0}^{*}/A_{r1}^{2}$ ($\beta_{2}=A_{r2}^{*}/A_{r1}^{2}$). Note that the data processing method was not used for the simulation data. The experimental and numerical $\beta_{0}$ and $\beta_{2}$ are shown in Figure 6a,b, respectively. The subgraph in Figure 6a is the locally enlarged image of experimental $\beta_{2}$ by narrowing the scale range of vertical direction. We can see that $\beta_{0}$ is strong and linearly proportional to the propagation distance. It grows without the limitation of phase-velocity matching. On the other hand, $\beta_{2}$ is weak and has a maximum cumulative propagation distance ($L_{n}$) due to the π effect caused by the slight deterioration of phase-velocity matching condition ($L_{n}=$ $2\pi /(k_{n}^{*}-2k)$ for $k_{n}^{*}\neq 2k$ [17,28]. And as shown in Figure 1d, the condition of phase-velocity matching is not strictly satisfied, when considering S0 (400 kHz) as the second harmonic of the primary mode. This phase-velocity mismatching ($k_{n}^{*}\neq 2k$) may cause a periodic fluctuation of the second harmonic. The similar experimental results of the second harmonic have been well documented in the literature [17]. $\beta_{0}$ of the zero-frequency mode is much stronger than $\beta_{2}$ of the second harmonic at all signal sampling points. To investigate the robustness of $\beta_{0}$, different frequencies of experimental excitation signals, for example, 250 and 300 kHz are considered and the similar results are obtained shown in Figure 7. β parameters are also presented for other frequencies in literature [39] and the numerical results of literature [39] are consistent with our theoretical predictions. It is confirmed that the zero-frequency mode can be utilized for evaluating the early-stage material nonlinearity.

**Figure 8:**
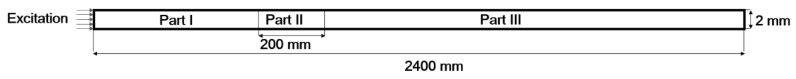
The model of monitor the local damage material nonlinearity.

**Figure 9:**
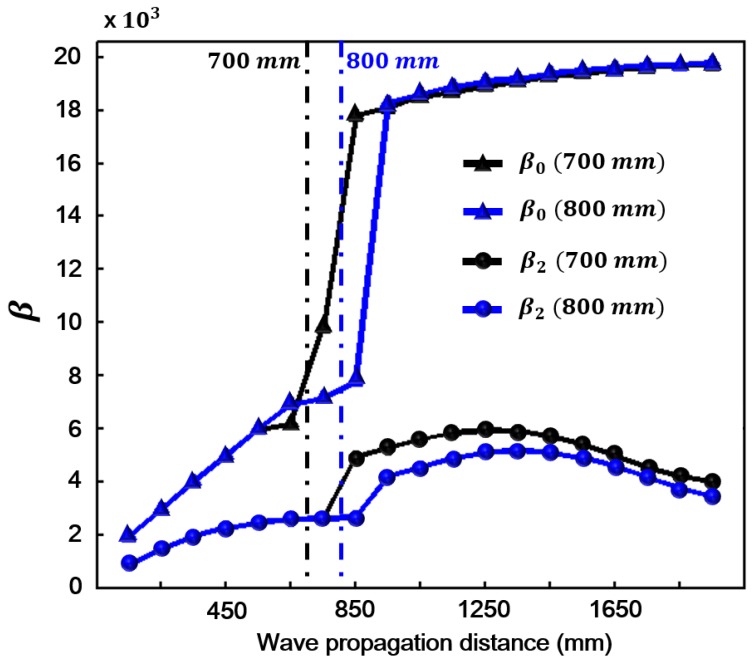
Acoustic nonlinearity parameter. β, a measure of material nonlinearity, plotted as a function of propagation distance for zero-frequency mode ($\beta_{0}$ marked by triangle) and the second harmonic ($\beta_{2}$ marked by circle).

To verify that $\beta_{0}$ can be used to monitor local damage, a simulation model as shown in Figure 8 is built. The material properties of Parts I and III are shown in Table 1 and the material properties of Part II is shown in Table 2. It can be noted that the three-order elastic coefficients in Table 2 is much larger than the ones in Table 1. Thus, it can be considered that the model contains the local fatigue damage in Part II. The width of Part II is 200 mm. We consider two locations of Part II: 700 and 800 mm away from the left end of the model, to investigate the change of $\beta_{0}$. The same nineteen signal detection points with the ones in Section 4 are considered. Figure 9 shows the simulation results of $\beta_{0}$ and $\beta_{2}$ for comparison. It can be seen that both $\beta_{0}$ and $\beta_{2}$ have a jump when waves propagate through the region of Part II and $\beta_{0}$ is much more sensitive to local change of the material nonlinearity. Therefore, the zero-frequency mode can be used as an effective efficient evaluation index for the early-stage material nonlinearity.

## 6. Conclusions

In conclusion, we present theoretical analysis and experimental and simulation results to demonstrate that the symmetric zero-frequency mode is effective for evaluating the early-stage material nonlinearity. Compared with the conventional techniques based on the second or higher-order harmonics of Lamb waves, the zero-frequency mode has many advantages, such as strong signals, easy accumulation, long-range inspection, arbitrary excitation frequency and no requirement of phase-velocity matching.

Theoretically, besides S0, other wave modes, for example, S1, A0, A1, etc. (Figure 1d), can also be used as the primary wave and yet only the symmetric zero-frequency mode can be generated due to the requirement of the non-zero power flux. The above method of using the symmetric zero-frequency mode is also valid for the case of using broad-band signals as the primary waves, which can be conveniently and inexpensively excited. Besides the weak material nonlinearity, we have also identified that the zero-frequency mode can be induced by a micro crack in an elastic plate, leading to the possibility of using the zero-frequency mode to detect and evaluate the micro crack or numerous distributed micro cracks. The above issues need to be confirmed with more experimental evidences in future. Nevertheless, the results presented in this work support that the zero-frequency mode can be used as an effective efficient evaluation index for the early-stage material nonlinearity.

## Figures and Tables

**Table 1:** Material properties of Al.

ρ (kg/m^3^)	λ (GPa)	μ (GPa)	A(GPa)	B(GPa)	C(GPa)
2704	70.3	26.96	−4160	−1310	−1505

**Table 2:** Material properties of Al in Part II.

ρ (kg/m^3^)	λ (GPa)	μ (GPa)	A(GPa)	B(GPa)	C(GPa)
2704	70.3	26.96	−41600	−13100	−15050
